# Confirmatory validation of the french version of the Duchenne Muscular Dystrophy module of the pediatric quality of life inventory (PedsQL^TM^3.0DMD*fv*)

**DOI:** 10.1186/s12887-023-04153-4

**Published:** 2023-11-15

**Authors:** Elisabeth Wallach, Virginie Ehlinger, Maelle Biotteau, Ulrike Walther-Louvier, Yann Péréon, Carole Vuillerot, Stephanie Fontaine, Pascal Sabouraud, Caroline Espil-Taris, Jean-Marie Cuisset, Vincent Laugel, Eloïse Baudou, Catherine Arnaud, Claude Cances

**Affiliations:** 1 Neuropediatric Department, Toulouse-Purpan University Hospital, Toulouse, France; 2 UMR 1295 CERPOP, Toulouse University, Inserm, University Toulouse III Paul Sabatier, Toulouse, France; 3 ToNIC, Toulouse NeuroImaging Center, University of Toulouse, Inserm, UPS, Toulouse, France; 4 CHU Montpellier, Service de Neuropédiatrie, Centre de Référence Maladies Neuromusculaires AOC, Montpellier, France; 5 Reference Centre for Neuromuscular Diseases AOC, Filnemus, Euro-NMD, Hôtel-Dieu, CHU Nantes, Nantes, France; 6 Hospices Civils de Lyon, Hôpital Femme-Mère-Enfant, L’Escale, Service de Médecine Physique et de Réadaptation Pédiatrique, Bron, France; 7 NeuroMyogen Institute, CNRS UMR 5310 - INSERM U1217, University of Lyon, Lyon, France; 8 Department of Paediatrics, French Reference Center for Neuromuscular Diseases, American Memorial Hospital, Reims University Hospital Center, Reims, France; 9 CHU Pellegrin, Service de neuropédiatrie, Centre de Référence Maladies Neuromusculaires AOC, Bordeaux, France; 10 Reference Centre for Neuromuscular Diseases Nord/Est/Ile-de-France, CHU Lille, Lille, France; 11 Department of Pediatric Neurology, CHU Lille, Lille, France; 12 Unité de neuropédiatrie et CIC pédiatrique, Hôpitaux Universitaires de Strasbourg, Strasbourg, France; 13 Clinical Epidemiology Unit, University Hospital, Toulouse, France

**Keywords:** Neuromuscular disorder, Duchenne Muscular Dystrophy, Pediatric Quality of Life (PedsQL)

## Abstract

Duchenne Muscular Dystrophy (DMD) is a neuromuscular disease that inevitably leads to total loss of autonomy. The new therapeutic strategies aim to both improve survival and optimise quality of life. Evaluating quality of life is nevertheless a major challenge. No DMD-specific quality of life scale to exists in French. We therefore produced a French translation of the English Duchenne Muscular Dystrophy module of the Pediatric Quality of Life Inventory (PedsQL^TM^DMD) following international recommendations. The study objective was to carry out a confirmatory validation of the French version of the PedsQL^TM^DMD for paediatric patients with DMD, using French multicentre descriptive cross-sectional data. The sample consisted of 107 patients. Internal consistency was acceptable for proxy-assessments, with Cronbach's alpha coefficients above 0.70, except for the *Treatment* dimension. For self-assessments, internal consistency was acceptable only for the *Daily Activities* dimension. Our results showed poor metric qualities for the French version of the PedsQL^TM^DMD based on a sample of about 100 children, but these results remained consistent with those of the original validation. This confirms the interest of its use in clinical practice.

## Background

Duchenne Muscular Dystrophy (DMD) is the most common progressive muscular dystrophy in children, with an incidence of 1/3,300 male births [1]. It is an inherited disease linked to the X chromosome resulting from pathogenic mutations in the *DMD* gene, which encodes a membrane cytoskeletal protein, dystrophin. DMD shows clinical motor, cardiac, respiratory and cognitive heterogeneity. The diagnosis is suggested by the following characteristic triad: progressive myopathic syndrome, Creatine Kinase increase, and pathological patterns of dystrophy. The development of the disease is progressive. Loss of walking ability inevitably occurs between the ages of 7 and 13 years, on average. Cognitive impairment is possible but varies between patients [2–4]. The course of DMD is marked by restrictive respiratory failure, cardiomyopathy, disorders of the static spine and nutritional difficulties [5, 6]. To date, there is no curative treatment, although coordinated multidisciplinary supportive care improves survival (adding about 15 to 20 years) [5–8]. The new therapeutic strategies, both current and future, aim to correct the primary genetic defect and thus limit the metabolic consequences and their functional impact. All these treatments aim to improve survival and optimise Quality of Life (QoL) [8].

QoL combines objective (socioeconomic), subjective (feelings of well-being), physical (chronic pathology) and cognitive factors. It is a measurable health indicator, which can be used as an outcome measurement in treatment trials or to assist decision-making in everyday medical practice. It is generally assessed using either generic scales or specific scales, through self- and/or proxy-assessments [9].

The evaluation of QoL in paediatrics requires the adaptation of factors such as age, possible activities, and family context. Those responsible for the child’s health (parents, caregivers, doctors) are directly involved in caregiving [9, 10].

**Fig. 1:**
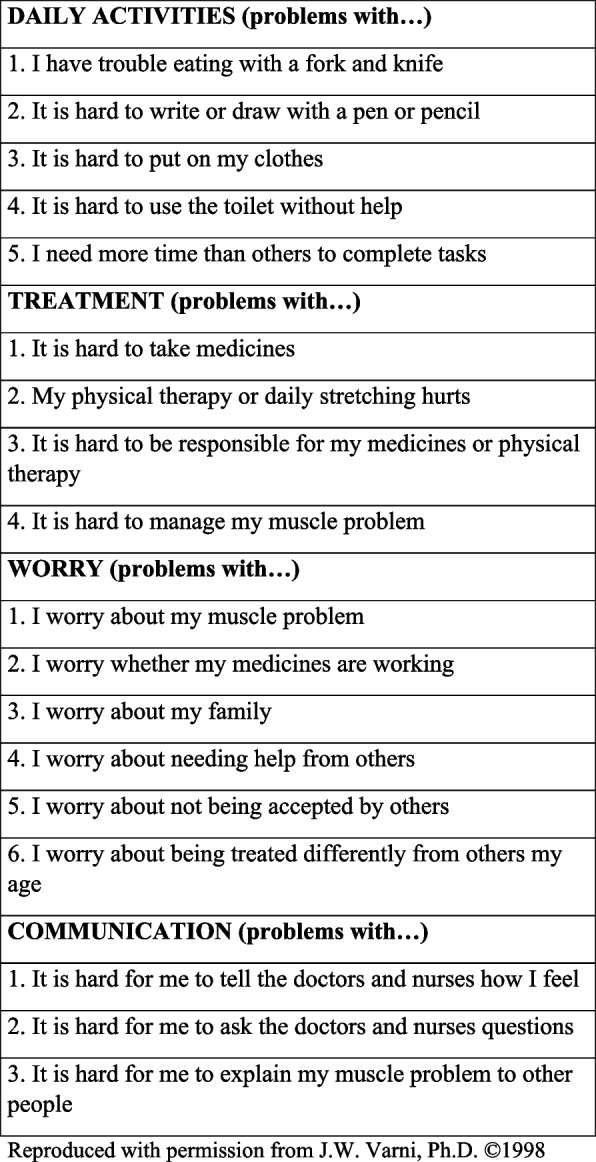
English-language version of the PedsQL^TM^DMD

Given the specific features of DMD, it would be useful to have a specific scale in French. Currently, the Duchenne Muscular Dystrophy module of the Pediatric Quality of Life Inventory (PedsQL^TM^DMD) is one of the most widely used [11]. The scale was validated in English by Uzark et al. in 2012 (Fig. 1) [12] and in other languages, for example Chinese [13] and Thai [14]. The English version of PedsQL^TM^DMD was previously translated into French by our team in accordance with international recommendations and established guidelines.

Forward translation (two independent bilingual, one French-mother-tongue and another English-mother-tongue, blinded translators) and back translation (one bilingual translator) were performed. Each step was reported in detail and submitted to the author of the original scale for agreement [15].

The objective of this study was to carry out a confirmatory validation of the French version (*fv*) of the DMD module of the PedsQL™ (PedsQL^TM^DMD*fv*) and document its metric properties in paediatric patients with DMD.

## Materials and methods

### PedsQL^TM^DMD’ scale

The PedsQL^TM^DMD targets three age groups: 5 to 7 years old, 8 to 12 years old, and 13 to 18 years old. It is available in two versions: proxy- and self-reports. The 18 items are divided into four dimensions: *Daily Activities*, *Treatment*, *Worry*, and *Communication*. Items are scored from 0 to 4 corresponding to ‘Never’, ‘Almost Never’, ‘Sometimes’, ‘Often’, and ‘Almost Always’. Dimension scores and a total score can be calculated and vary from 0 to 100. The closer the score is to 100, the better the QoL perceived by the child or caregiver. The recall period is one month.

The English version is used to assess QoL in clinical trials and daily clinical practice, given its robust internal consistency (close to 0.8) and adaptability to a wide range of ages.

### Design and population

For this French multicentre descriptive cross-sectional study, children and their parents were included during a multidisciplinary consultation at their reference centre, between July 2018 and January 2019. Follow-up of the patients included in the study is being provided at the paediatric departments of the French Reference Centres for Neuromuscular Diseases in Toulouse, Bordeaux, Montpellier, Lyon, Reims, Strasbourg, Lille and Nantes.

The inclusion criteria were as follows: male children aged 5 to 18 years, carriers of genotyped DMD, registered in the BaMaRa database (national Rare Disease registry), and whose parents or guardians did not object to the child’s participation. The non-inclusion criteria were: inability to understand the questions, lack of parental authority, antidepressant or anti-psychotic treatment, non-French-speaking child and/or parents, and girl with DMD.

### Data collection

At a biannual multidisciplinary consultation, the PedsQL^TM^3.0DMD*fv* was presented to the child and one or both parents by the child’s doctor or the psychologist of the department. Specifically, children over 8 years rated their own QoL and the parents of children of all ages also rated their child’s QoL. The questionnaires were completed separately in different rooms so that parents and children were not aware of each other’s responses. However, the child or the parent could consult the doctor/psychologist if they did not understand a question. The completion time was 15 to 20 min.

The score for each dimension of the DMD module was calculated as the average of the component items for the dimension. The response options were coded as follows: Never: 100 points, Almost Never: 75 points, Sometimes: 50 points, Often: 25 points, and Almost Always: 0 points, according to the authors’ instructions.

The following routine data were extracted directly from each patient’s medical records at the together with motor function (assessed by the *Motor Function Measure*/MFM [16], wheelchair use, and age at loss of walking ability); respiratory function (pulmonary function testing: forced vital capacity, respiratory support, type of respiratory support); cardiac function (left ventricular ejection fraction); nutritional status (weight, height, BMI, nutritional support by gastrostomy); school status (ordinary schooling, personal assistance, specialised schooling); and current drug treatment (corticosteroids, ACE inhibitors).

### Statistical analysis

The response distributions of individual items, rates of floor and ceiling effects, are reported as frequencies and percentages, and subscale scores as mean ± standard deviation (SD), median and interquartile range, and minimum and maximum. The feasibility of the module was assessed using the percentage of missing data.

The internal consistency of each subscale was measured by Cronbach’s alpha coefficient in the whole sample and by age, with values above 0.70 considered as acceptable.

Construct validity was then examined. We investigated the structural validity of the module using a multi-trait scaling approach [17]. Polychoric correlations between individual items were estimated [18, 19]. Each item’s convergent validity was considered satisfactory if its correlation between with the other items within the same subscale and with its subscale score omitting that item (item-total correlation corrected for overlap) exceeded ≥ 0.40. Moreover, an item’s discriminant validity was judged sufficient if the item was more correlated with its own subscale than with the others.

Inter-subscale correlations were estimated. We used the Pearson correlation coefficient when the linearity of the association was demonstrated, or otherwise the Spearman rank correlation coefficient. We expected correlations between 0.30 and 0.70.

Construct validity was also assessed by the known-groups method. We anticipated lower patient and proxy PedsQL^TM^3.0DMD subscale scores with increasing DMD severity, defined as greater age, loss of walking ability, ventilation, Conversion Enzyme Inhibitor therapy, corticosteroid therapy. Severe DMD was also defined as non-outpatient and receiving ventilation.

Finally, agreement between the self‐assessed and proxy scores was measured by the intraclass correlation coefficient (ICC) estimated in a two-way mixed-effects model (absolute agreement) [20], and visually inspected using Bland and Altman plots. We used the same thresholds as Uzark et al. to interpret the ICC values, namely: ICC < 0.40 indicated poor-to-fair agreement; 0.41–0.60, moderate agreement; 0.61–0.80, good agreement; and 0.81–1.00, excellent agreement. Agreement between patients and their parents at the item level was evaluated through the proportion of observed agreement and the weighted kappa coefficient with quadratic weighting [21].

Analyses were performed with Stata14 and the R_*v3.5.2Polycor* package [22].

### Ethics

The study was approved by the local Institutional Review Board (Southeast protection of individuals committee V) on 4 July 2018 (ID-RCB: 2018-A00895-50). An information note was given to the parents and an age-appropriate one to the child.

## Results

### Description of the sample, items and dimension scores

The analysis sample consisted of 107 patients: 16 in the 4–7 age group, 53 in the 8–11 age group, 38 in the 12–18 age group. The clinical characteristics according to age are set out in Table 1.

**Table 1:** Patient characteristics by age group

	**Age group**			
	**4–7 yrs**	**8–11 yrs**	**12–18 yrs**	**Total**
	**(*N* = 16)**	**(*N* = 53)**	**(*N* = 38)**	**(*N* = 107)**
**Variable**	**n(%)**	**n(%)**	**n(%)**	**n(%)**
**Clinical diagnosis**				
Deletion	11 (84.6)	31 (62.0)	24 (64.9)	66 (66.0)
Duplication	1 (7.7)	8 (16.0)	3 (8.1)	12 (12.0)
Point mutation	1 (7.7)	11 (22.0)	10 (27.0)	22 (22.0)
**Walking ability acquired**				
No	0 (0.0)	0 (0.0)	0 (0.0)	0 (0.0)
Yes	15 (100.0)	53 (100.0)	38 (100.0)	106 (100.0)
N missing	1	0	0	
**Loss of walking ability**				
No	15 (100.0)	24 (46.2)	0 (0.0)	39 (37.1)
Yes	0 (0.0)	28 (53.8)	38 (100.0)	66 (62.9)
N missing	1	1	0	
**Severity: not walking and ventilated**				
No	16 (100.0)	50 (94.3)	29 (76.3)	95 (88.8)
Yes	0 (0.0)	3 (5.7)	9 (23.7)	12 (11.2)
N missing	0	0	0	
**Medication treatment: corticosteroids**				
No	8 (57.1)	10 (20.0)	25 (65.8)	43 (42.2)
Yes	6 (42.9)	40 (80.0)	13 (34.2)	59 (57.8)
N missing	2	3	0	

The total MFM score ranged from 1 to 98.9% with a median of 57%. Among the 12 patients receiving non-invasive ventilation, ventilation was intermittent in 11 cases and continuous in one case. Nine of these patients were in the 12–18 age group. None were in the 5–7 age group.

For these 107 patients, we were able to use 89 self-reports (8–18 years) and 99 proxy-reports (5–18 years): 61 from the mother, and 38 from both parents together. The analysis of agreement between the responses to the self and proxy-assessments included 81 child-parent pairs: 49 pairs in the 8–11 age group and 32 pairs in the 12–18 age group.

Table 2 sets out the distribution of scores for the four dimensions: *Daily activities, Treatment*, *Worry*, and *Communication*. Parents’ scores ranged from 0 to 100 for all four dimensions. The self-report scores covered a narrower range, particularly for the *Worry* dimension (minimum score: 41.7 points). The variability of the scores (measured by standard deviation) appears to be lower for the self-report scores in general, especially for the *Worry* dimension.

**Table 2:** Univariate description of the self- and proxy-assessment scores on the PedsQL^TM^3.0DMD*fv*

**Dimension**	**Self-assessment**				**Proxy-assessment**			
	**N**	**Min–Max**	**Med (IQR)**	**Mean (SD)**	**N**	**Min–Max**	**Med (IQR)**	**Mean (SD)**
Daily Activities	89	0.0 – 100.0	45.0 (35.0 – 65.0)	49.3 (26.3)	99	0.0 – 100.0	40.0 (20.0 – 60.0)	41.3 (25.2)
Treatment	89	12.5 – 100.0	62.5 (56.3 – 81.3)	67.3 (19.0)	98	0.0 – 100.0	62.5 (43.8 – 81.3)	62.0 (23.1)
Worry	89	41.7 – 100.0	83.3 (70.8 – 91.7)	80.9 (14.9)	99	0.0 – 100.0	58.3 (41.7 – 79.2)	58.0 (24.0)
Communication	89	0.0 – 100.0	58.3 (41.7 – 83.3)	58.2 (27.6)	99	0.0 – 100.0	50.0 (25.0 – 75.0)	48.0 (32.5)

Non-responses to items were very rare. We observed no marked ceiling or floor effect.

### Fidelity of the PedsQL^TM^3.0DMD*fv*

Internal consistency was acceptable for the proxy-assessments (Cronbach's alpha > 0.70) with the exeption of the *Treatment* dimension. For the self-assessments, internal consistency was acceptable only for the *Daily Activities* dimension. It was insufficient for the other dimensions (Table 3). Although the estimates among patients with severe disease were highly imprecise due to small numbers, the findings remained unchanged when stratified by disease severity (data not shown).

**Table 3:** Internal consistency of the PedsQL^TM^3.0DMD*fv* Dimensions: Cronbach’s alpha

**Dimension**	**Child (self-assessments)**			**Proxy-assessments**			
	**Total**	**Group 8–11 yrs**	**Group 12–18 yrs**	**Total**	**Group 4–7 yrs**	**Group 8–11 yrs**	**Group 12–18 yrs**
	**(*n* = 89)**	**(*n* = 51)**	**(*n* = 38)**	**(*n* = 99)**	**(*n* = 16)**	**(*n* = 51)**	**(*n* = 32)**
**Daily Activities**							
Pearson	0.74	0.72	0.70	0.81	0.86	0.75	0.66
Polyc. + corr.cont	0.78	0.73	0.69	0.82	0.68	0.69	0.74
**Treatment**							
Pearson	0.43	0.41	0.51	0.68	0.79	0.53	0.76
Polyc. + corr.cont	0.50	0.51	0.44	0.70	0.69	0.52	0.70
**Worry**							
Pearson	0.52	0.58	0.43	0.83	0.87	0.84	0.76
Polyc. + corr.cont	0.72	0.77	0.68	0.85	†	0.83	0.64
**Communication**							
Pearson	0.66	0.60	0.68	0.87	0.85	0.85	0.90
Polyc. + corr.cont	0.69	0.56	0.66	0.89	0.75	0.84	0.81

Pearson: Cronbach alpha coefficient based on the Pearson correlation matrix

Polyc. + corr.cont: Cronbach alpha coefficient based on the polychoric correction matrix with correction for empty cells

† Not estimated, because one item had an empty category for the subgroup

Polychoric correlation coefficients showed a satisfactory convergent validity of each item for the self and proxy-assessments; the discriminant validity of each item was also satisfactory. Most of the correlations between the four dimensions of the self-assessments presented in Table 4 were moderate between 0.30 and 0.70. The correlations between the *Daily Activities* and *Worry* subscales were particularly weak. Notably, there was poor correlation between the *Communication* subscale and the other subscales. The correlations between scores for the parents on the four subscales were between 0.30 and 0.70, but weaker correlations were observed between the *Communication* subscale and the other subscales, especially for the evaluations concerning the 8- to 11-year-old patients (data not shown).

**Table 4:** Inter-correlations between self-evaluation scores on the PedsQL^TM^3.0DMD*fv*, *N* = 89

	**PedsQL**™** Dimensions**			
	**Daily Activities**	**Treatment**	**Worry**	**Communication**
**Group 8–11 yrs**				
Daily Activities	1.000	0.566	0.156 †	0.244
Treatment	0.566	1.000	0.531 †	0.294
Worry	0.156 †	0.531 †	1.000	0.263
Communication	0.244	0.294	0.263	1.000
**Group 12–18 yrs**				
Daily Activities	1.000	0.535	0.214 †	0.050
Treatment	0.535	1.000	0.297 †	0.151
Worry	0.214 †	0.297 †	1.000	0.339
Communication	0.050	0.151	0.339	1.000
**All ages combined**				
Daily Activities	1.000	0.555	0.198 †	0.054
Treatment	0.555	1.000	0.439 †	0.189
Worry	0.198 †	0.439 †	1.000	0.258
Communication	0.054	0.189	0.258	1.000

Pearson’s correlation coefficient are presented

† Spearman’s correlation coefficient is presented due to a non linear monotone association between scores

**Fig. 2:**
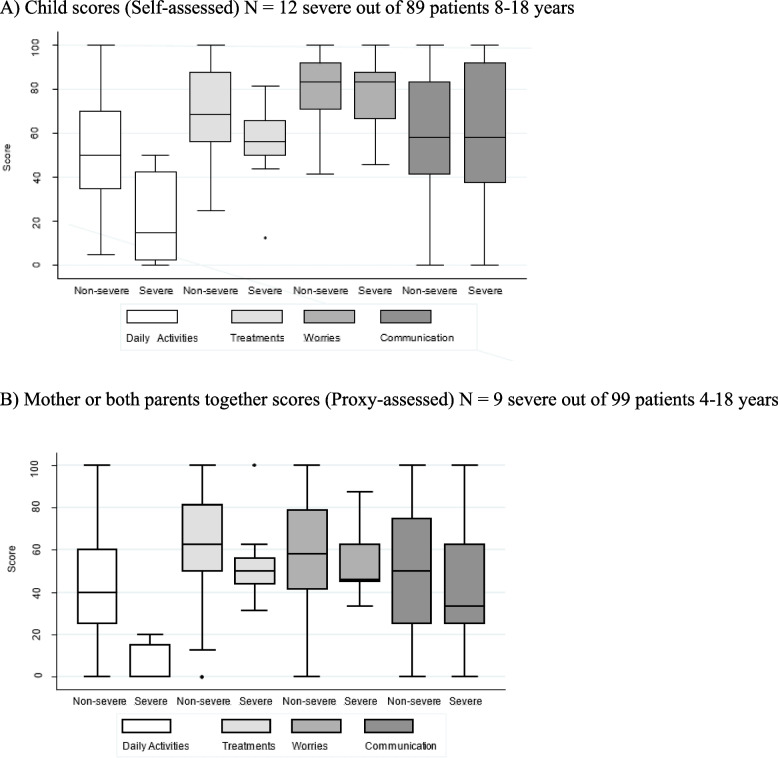
Distribution of scores (“Child” and “Mother”) according to DMD severity. **A** Child scores (Self-assessed) N = 12 severe out of 89 patients 8–18 years. **B** Mother or both parents together scores (Proxy-assessed) N = 9 severe out of 99 patients 4–18 years

To analyse the discriminant validity of the PedsQL^TM^3.0DMD*fv*, we assessed whether disease severity was associated with the PedsQL^TM^3.0DMD scores. Figure 2 presents “Child” and “Mother” scores as a function of “Severity”. The QoL score was significantly lower in the *Daily Activities* dimension for both the self- and proxy-assessments.

A total of 49 and 32 child-parent pairs were examined in the 8–11 and 12–18 age groups, respectively. Table 5 presents the difference (parent score-child score) between the children’s scores and those of their mothers or both parents together. The general trend indicated lower proxy scores than self-scores.

**Table 5:** Description of the differences between the PedsQL^TM^3.0DMD self-assessed and proxy-assessed scores, and the reliability indices between the self- and proxy-assessments

Domain	Self-assessment		Proxy-assessment		Mean difference		ICC 2-way mixed effects model
**Dimension**	**N**	**Mean (SD)**	**N**	**Mean (SD)**	**N**	**Mean (SD)**	**[95% CI]**
**All ages combined**							
Daily Activities	81	50.4 (26.0)	81	38.1 (24.2)	81	-12.3 (14.1)	0.75 [0.29–0.89]
Treatment	81	66.8 (19.1)	81	61.1 (23.2)	81	-5.7 (22.0)	0.45 [0.26–0.61]
Worry	81	81.2 (13.9)	81	56.5 (23.0)	81	-24.7 (23.0)	0.15 [-0.07–0.36]
Communication	81	56.9 (27.3)	81	46.5 (31.4)	81	-10.4 (36.3)	0.23 [0.02–0.42]
**Group: 8–11 yrs**							
Daily Activities	49	59.5 (24.8)	49	47.4 (24.1)	49	-12.0 (13.9)	0.75 [0.28–0.90]
Treatment	49	70.0 (18.8)	49	65.5 (18.7)	49	-4.5 (18.3)	0.51 [0.28–0.69]
Worry	49	82.3 (15.0)	49	57.2 (24.5)	49	-25.2 (23.3)	0.19 [-0.08–0.45]
Communication	49	52.4 (27.4)	49	44.6 (30.8)	49	-7.8 (38.9)	0.11 [-0.17–0.37]
**Group: 12–18 yrs**							
Daily Activities	32	36.6 (21.6)	32	23.8 (16.3)	32	-12.8 (14.5)	0.59 [0.08–0.82]
Treatment	32	62.0 (18.9)	32	54.5 (27.8)	32	-7.5 (27.0)	0.34 [0.01–0.61]
Worry	32	79.6 (12.0)	32	55.4 (20.8)	32	-24.1 (22.7)	0.05 [-0.11–0.27]
Communication	32	63.8 (26.1)	32	49.3 (32.6)	32	-14.5 (32.0)	0.38 [0.06–0.63]

The ICCs were low for the *Worry* and *Communication* subscales, indicating poor agreement in the parent–child dyads. They were low to moderate across age groups for the *Treatment* subscale, and fairly good for *Daily Activities*. This last result should be qualified, however, as the precision around the ICC estimate is specifically very low.

## Discussion

### Population

We obtained a representative sample in terms of diagnosis, with a predominance of deletions (66%), a 12% rate of duplications and a point mutation rate of 22% consistent with the literature [23]. The total MFM score varied from 1 to 98.90% (median of 57%) ranging from patients with less severe up to very severe motor impairment. Corticosteroids were administered to 57.8% of the population compared with 85% for the English-language validation, probably due to divergences between English and French language guidelines.

### Fidelity of the PedsQL^TM^3.0DMD*fv*: internal consistency

Our results showed good internal consistency for the parents’ scores, except for the *Treatment* dimension. For the children, internal consistency was acceptable only in the *Daily Activities* dimension, which provides objective information on everyday living. In the validation study of the original English-language version, with 200 parents and 117 children (i) the internal consistency was better, with Cronbach's alpha close to 0.8 (ii) the difference between the children’s and parents’ data was less significant with parents’ data showing higher Cronbach's alphas, (iii) the lowest Cronbach's alpha for both datasets was in *Treatment* dimension [12].

In our French version, the lower internal consistency for both children and parents’ scores -compared to validation study of the original English-language version- could probably suggested a misunderstanding of the several items. For example, children found difficult to understand the concept of *Treatment* in our initial translation process. We therefore replaced it by *Medicines and Physiotherapy*. In clinical practice, this item could be even more specific with the names of the medications or the number of tablets taken per day, which would help the children to give a more precise answer. Another explanation could be that the heterogeneity of our sample, ranging from less severe up to severe impairments, could warrant this finding. We therefore conducted an assessment by severity, which allowed us to examine the scale’s internal consistency separately in several subgroups. It remained imprecise due to the low numbers, but conclusions did not change: the internal consistency was insufficient for the children’s questionnaires and satisfactory for the parents’ questionnaires, with a similar distribution of scores.

Analysis of internal consistency and its sensitivity revealed good internal consistency, especially for the responses from the parents’ group and the severe group of children. This analysis was not performed in the original validation.

In our study, the differences between the children’s and parents’ findings were greater than in the validation study of the original English version, with the parents’ data showing higher Cronbach's alphas. The lowest Cronbach's alpha for both datasets was for the *Treatment* dimension [12]. The difference can be explained by a change in the wording of items in our French translation. Indeed, although identical for all ages in the children’s or the parents’ questionnaires, the items were formulated differently for the children and their parents. This difference was marked for item 4: "I am having trouble living with my illness [children]" and "Difficulties in managing his/her muscle disorder [parents]". However, the back translation was correct. This item 4 seems to be related more to the *Worry* dimension than to the *Treatment* dimension. However, it is ranked in *Treatment* dimension (in the original version and consequently in our French translation) which can be a little confusing.

Internal consistency was lower for the *Treatment* dimension for the parents’ assessments and was better for the *Daily Activities* dimension (which provides objective and representative information on everyday living) for the children’s assessments. Our results were consistent with the original English version where the lowest Cronbach's alpha for both datasets was in the *Treatment* dimension [12].

### Construct validity

First, the convergent validity was satisfactory for each item in the *Daily Activities* and *Communication* subscales, for both child and parent ratings. It was also good for the *Treatment* and *Worry* subscales, but only for the parents’ assessments. The correlation coefficients were higher in the *Daily Activities* dimension than in the other dimensions. These findings seem logical. The *Daily Activities* dimension reflects motor impairment and severity and is composed of objective items. The *Worry* and *Communication* dimensions are more subjective and are much more directly dependent on the child or the parents’ feelings, which makes probably them less reliable.

Second, several items from various dimensions were positively correlated with items of a different dimension. For example, item 3 of the *Treatment* dimension appeared to be more closely associated with the *Daily Activities* dimension sub-scores, which is consistent with the fact that the *Daily Activities* dimension is the more objective dimension and the one more closely linked to motor impairments.

The same pattern was found for item 4 of the *Treatment* dimension, which appeared to be associated with the *Daily Activities*, *Treatment*, and *Worry* dimensions. It is possible that this item could be too subjective in its interpretation. This analysis was not reported in the original validation article.

Third, most of the correlations between the questionnaire dimensions were moderate. The *Communication* dimension showed little correlation with the other dimensions. This raises the question of its relevance and therefore that of the notion of an overall QoL score. In the original validation, these correlations were not performed.

Finally, for the oldest children with severe DMD, the QoL reported by the children and their parents was significantly poorer for the *Daily Activities* dimension. For the other dimensions, the results were not significant. Our result was consistent with the original article where, for the *Daily Activities* dimension, QoL was significantly better for the 8–12-year-old children than for the 13–18-year-olds, both for children and parents. This previous result was reinforced by a significant difference -both for children and parents- between children needing mobility aids and those moving without assistance: QoL was deemed to be better for those who didn’t use mobility aids or could walk [12]. Similarly, Davis et al., in their 2010 study to validate the neuromuscular module for the DMD population, also found that children and parents reported a poorer QoL compared to a healthy population, most importantly in the physical dimension. QoL was significantly better in patients who did not use a wheelchair or very rarely compared to those who used one all the time [24]. Thus, as with previous studies, ours clearly demonstrated the progressive chronic condition component of DMD, leading to irreversibly reduced physical condition, mobility and autonomy, and consequently a gradual decline in QoL.

### Agreement between self- and proxy-assessments

The overall trend was that proxy-assessments scores (parents) were lower than self-assessments (children): parents rated their child's QoL worse than their children thought. The difference was more marked for subjective items corresponding to feelings (*Worry* and *Communication* dimensions) compared to more objective items (*Treatment* and *Daily Activities* dimensions). For the *Worry* dimension, the correlation was better between proxy-assessment (parent) and self-assessment (child) scores when the child's QoL was good. Our findings showed the same trend as the original study: the ICC was between 0.61–0.80 for *Daily Activities*, which indicated good agreement, and moderate For *Treatment* and *Worry (*ICC = 0.41–0.60). The weakest ICC was for the *Communication* dimension [12]. This perception gap-both in original and in our translation- may be linked to the considerable difficulty parents had in understanding their position when replying to the questionnaire. Several parents thought that they had to assess their own QoL.

Our French version of the scale showed poor agreement between the parents and children, similar to the findings on the original English scale. The agreement was better for objective functions (activities of daily living) and lower for more subjective functions (Worry, emotions). Children appeared less worried than their parents in both versions. This result is consistent with literature on this subject and refers to the "disability paradox" or "well-being paradox" [25, 26], a process of adapting to changes in health and accommodation to illness. Children think they have a better QoL than their parents, because they have become accustomed to their diseases. This is the only life they know and they rate their QoL as relatively good, and perceive their well-being differently. Reference can also be made to the "coping strategy", an adaptation strategy that helps protect against the adverse effects of disease. Explaining the “disability paradox” to families might therefore give them a more positive view of their child's experience.

Another explanation to explain this difference is that parents have different perspectives on their child's illness, and they probably feel more anxious about the future. They adjust their perceptions based on their knowledge of the natural course of the disease and think negatively about the future [27]. Also, parents are often overwhelmed by the care and their negative perceptions can also be explained by the "burden” of being an informal caregiver [28]. DMD leads to intense and prolonged family involvement, leading to physical, psychological and financial consequences [29]. It will be probably necessary in the future to assess the parents’ QoL -or at least to collect data on their mental health or their own difficulties- in order to interpret the results more precisely.

## Conclusion

Based on a representative sample (100 patients), we were able to demonsrate the useful metric qualities of the French version of the PedsQL^TM^3.0DMD. Our results, which are consistent with those of the original version, validate the relevance and use of the PedsQL^TM^3.0DMD and its use in clinical practice. We would therefore propose that the analysis of QoL in these patients be undertaken on a broader scale, or even systematically. We would also suggest that there is a need to take this factor into account in future therapeutic trials.

However, assessing the QoL of DMD' patients appears to be a complex task. PedsQL^TM^3.0DMD is a useful tool but several limitations have been reported. it seems useful to propose a more comprehensive tool that takes into account, for example, the affected muscles, the degree of severity, the level of pain and fatigue of the children, etc. Furthermore, the creation of a cognitive level-adjusted scale would appear to be suitable. In fact, we were unable to include all of our patients due to moderate to severe cognitive difficulties that prevented their understanding of the scale. If we could enlarge our population sample using a cognitive -level-adjusted scale, one example being the SOLE questionnaire [30], it would be useful to revise our data according to IQ level in the different subgroups to obtain more representative insights.

## Data Availability

The datasets used and/or analysed during the current study are available from the corresponding author on reasonable request.

## Abbreviations

DMD: Duchenne Muscular Dystrophy
ICC: Intraclass Correlation Coefficient
MFM: Motor Function Measure
QoL: Quality of Life
PedsQL: Pediatric Quality of Life
SD: Standard Deviation
